# Age-Related Differences in Motor Skill Transfer with Brief Memory Reactivation

**DOI:** 10.3390/brainsci14010065

**Published:** 2024-01-09

**Authors:** Kylie B. Tomlin, Brian P. Johnson, Kelly P. Westlake

**Affiliations:** 1 Department of Physical Therapy and Rehabilitation Science, University of Maryland School of Medicine, Baltimore, MD 21201, USA; 2 Department of Occupational Therapy, School of Medicine, Washington University in St. Louis, St. Louis, MO 63110, USA

**Keywords:** motor learning, memory, motor skills, memory reactivation, intermanual transfer, aging

## Abstract

Motor memories can be strengthened through online practice and offline consolidation. Offline consolidation involves the stabilization of memory traces in post-practice periods. Following initial consolidation of a motor memory, subsequent practice of the motor skill can lead to reactivation and reconsolidation of the memory trace. The length of motor memory reactivation may influence motor learning outcomes; for example, brief, as opposed to long, practice of a previously learned motor skill appears to optimize intermanual transfer in healthy young adults. However, the influence of aging on reactivation-based motor learning has been scarcely explored. Here, the effects of brief and long motor memory reactivation schedules on the retention and intermanual transfer of a visuomotor tracing task are explored in healthy older adults. Forty older adults practiced a virtual star-tracing task either three (“brief reactivation”) or ten (“long reactivation”) times per session over a two-week period. Comparison with a previously reported group of younger adults revealed significant age-related differences in the effect of the motor memory reactivation schedule on the intermanual transfer of the motor task. In older adults, unlike younger adults, no significant between-group differences were found by practice condition in the speed, accuracy, or skill of intermanual task transfer. That is, motor task transfer in healthy younger, but not older, adults appears to benefit from brief memory reactivation. These results support the use of age-specific motor training approaches and may inform motor practice scheduling, with possible implications for physical rehabilitation, sport, and music.

## 1. Introduction

Classic theories of memory formation, including Müller and Pilzecker’s (1900) consolidation model and Atkinson and Shiffrin’s (1968) memory model, have been applied to motor skill learning to describe processes of motor memory formation and stabilization. These models suggest that memory development occurs in a series of stages, during which encoded representations are briefly stored in short-term memory and then progressively strengthened, or consolidated, over time to a more stable state in the long-term memory [1,2,3]. More recently, it has been theorized that consolidated memories can be updated to adjust to new contexts or demands through processes of reconsolidation [4,5,6,7].

Re-exposure to a stimulus may temporarily destabilize, or reactivate, a previously consolidated memory, allowing the memory trace to be modified and subsequently restabilized, or reconsolidated, in long-term storage [4,5,6,7]. Early evidence on reactivation–reconsolidation processes was provided by Misanin and colleagues (1968) who showed, in an animal model, that the interference of a stable memory could only occur following the re-introduction of a previously conditioned stimulus [4]. Support has since emerged for the involvement of reactivation and reconsolidation processes in the interference [6] and enhancement [7] of human motor performance. Wymbs et al. (2016), for instance, suggest that existing motor memories can be strengthened through practice-induced memory reactivation, resulting in improved motor behavioral outcomes. Practice-induced memory reactivation may have practical implications in sport, music, and physical rehabilitation contexts in which motor skills are repeatedly rehearsed and motor memories may be strengthened through iterative reactivation–reconsolidation cycles.

Whether for the purposes of independent learning or rehabilitation, a motor skill is often practiced recurrently. When a motor skill is practiced, a motor memory trace is formed. This motor memory can later be recalled for use on the same task, that is retention, or for use on a similar but distinct motor task, as in the case of motor skill transfer or generalization. Motor retention is necessary for delayed application of an acquired skill and for iterative skill progression over multi-session practice. Motor transfer is important for behavioral efficiency through the versatility of skill application in novel contexts; moreover, transfer may have particular relevance for physical rehabilitation as the environment in which learned motor skills are applied (e.g., home) is often different from where they are practiced (e.g., clinic).

During motor practice, neural representations of motor behaviors may be reactivated briefly, through short exposure, or over a larger number of repetitions. In physical rehabilitation, for example, individuals often complete more than 20 repetitions of a specific motor behavior within a single therapy session and may repeat this training over multiple sessions each week [8,9]. Because common motor training approaches likely involve repetitive reactivation–reconsolidation processes, investigation on approaches to optimize reactivation-based motor learning may maximize functional motor outcomes including skill retention and transfer [10].

Practice-induced reactivation and reconsolidation processes may be influenced by several factors including the presence of prediction errors, timing of stimulus presentation, and duration of stimulus re-exposure [11,12]. De Beukelaar and colleagues (2014), for example, demonstrated that the length of motor memory reactivation had a significant influence on the retention of a key press task in the presence of interference. These authors concluded that brief reactivation (<60 s) resulted in a motor memory being more labile to change through interference as compared to long reactivation (>60 s) in healthy young adults [12]. It seems reasonable that the length of motor memory reactivation may also influence practice-induced strengthening of motor performance. Recently, it was shown that brief, continuous, and error-free reactivations of a motor sequence memory improve motor outcomes, including transfer of the sequence task, in healthy young-to-middle-aged adults [13]. Relatedly, our group demonstrated that the transfer of a visuomotor skill to the contralateral upper extremity (i.e., intermanual transfer) is optimized following brief, but not long, motor memory reactivation in a healthy young adult population [14]. Although the evidence on factors influencing reactivation–reconsolidation processes is expanding, the role of age in reactivation-based motor learning is not well understood. Accordingly, the present study focuses on reactivation scheduling in an older adult sample.

Age-related differences in the effects of practice-induced reactivation may be expected based on demonstrated impairments in sleep-dependent consolation in older compared to younger adults [14]. Weakened sleep-dependent consolidation observed in healthy aging is often attributed to age-related alterations in sleep quantity and quality; specifically, reduced duration of slow-wave sleep and frequency and amplitude of sleep spindles may contribute to learning and memory differences in aging [15,16,17]. Impaired consolidation and retention of motor skills in healthy older compared to younger adults have been demonstrated through behavioral [18,19] and neural monitoring [20] methods. Given these impairments in consolidation, and correspondingly reconsolidation, we suspect older adults may have limitations in reactivation-based enhancement of motor retention and transfer.

The purpose of this study is to compare the effect of brief and long reactivation schedules on retention and intermanual transfer of a complex upper extremity virtual tracing task in healthy older adults. Here, we define a complex motor task as one that involves multi-joint movements over multiple directions with an opportunity for the integration of online sensorimotor feedback. The virtual tracing task, specifically, was selected to support age cohort comparisons, building on the tasks’ use in prior research with younger adults [14]. We define intermanual transfer as task performance using the untrained upper extremity contralateral to the trained upper extremity. We hypothesize that in healthy older adults, similar to healthy younger adults, brief compared to long reactivation of a previously learned motor task will result in similar skill retention and superior intermanual skill transfer measured at two weeks from initial learning. However, we hypothesize that, regardless of reactivation length, older adults will demonstrate overall reduced motor skill transfer compared to younger ones.

## 2. Materials and Methods

This study was approved by the University Institutional Review Board and all subjects provided consent to participate. Forty healthy older adults between 65 and 84 years old were recruited through convenience sampling. A priori power analysis, informed by Johnson et al.’s (2022) comparison of reactivation-based motor learning schedules in young adults, was conducted using G*Power 3.1 (G*Power, RRID:SCR_013726) to determine minimum sample size [14]. The results indicated n = 36 was required to achieve 80% power for detecting a large effect size at a significance α = 0.05. Inclusion criteria included regular access to a computer with a handheld mouse, at least one previous experience using an online meeting platform, clear right-hand dominance as indicated by an Edinburgh handedness index (Oldfield, 1971) of greater than or equal to 75 (R) [21], score within normal range on Saint Louis University Mental Status Exam (score ≥ 27) [22] and the Mini-Mental State Examination (score ≥ 24) [23], and no self-reported significant psychologic, neurologic, musculoskeletal, or other medical conditions that would impair motor skill participation. Of note, prior use of an online meeting platform was selected as a criterion to promote comfortability with the motor practice environment and procedures. Additionally, clear hand dominance was selected as a criterion to avoid potential effects of ambidexterity on intermanual transfer (primary outcome). Subject demographic information is outlined in Table 1.

### 2.1. Motor Task

**Figure 1:**
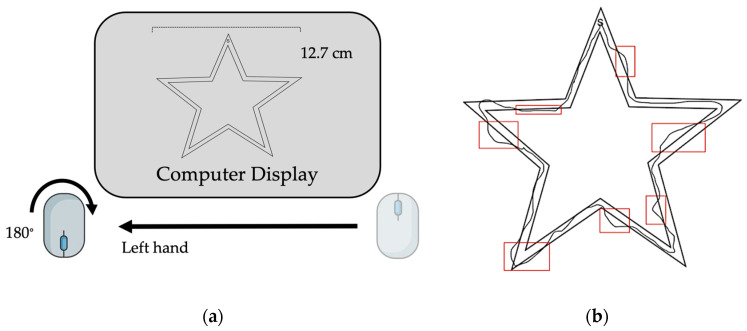
Task design. (**a**) Computer set-up with inverted handheld mouse positioned on non-dominant (left) side. (**b**) Example of completed trial attempt with error indicated.

The motor task used in this study has been previously reported by Johnson et al. (2022) [14]. The task involved the use of a handheld computer mouse rotated 180° from the upright position in the non-dominant (left) hand to trace a virtual image of a star (Figure 1a,b). The non-dominant hand was chosen for use to mitigate ceiling effects of learning the motor task. Subjects were instructed to trace the image clockwise as quickly and accurately as possible following a “3, 2, 1, Go” countdown. Sessions took place using Zoom^TM^ videoconferencing software (Version 5.14.2 (14578), Zoom Communications Inc., 2016, San Jose, CA, USA). Image width was fixed to 12.7 cm and cursor diameter was controlled between subjects. Subjects performed the task during seven virtual sessions over a two-week period (Table 2). On the first session, all subjects performed nine acquisition trials and were subsequently randomized into a three-trials (“brief reactivation”) or ten-trials (“long reactivation”)-per-session practice group. Over the five following sessions, subjects trained on the motor task according to group allocation with an intertrial interval of 30 s and an intersession interval range of 24–72 h (Table 2). At the seventh session, two weeks after initial learning, skill retention and intermanual transfer were assessed through the performance of three trials with the trained, non-dominant upper extremity (i.e., retention) and three trials with the untrained, dominant upper extremity (i.e., intermanual transfer).

### 2.2. Outcomes

Performance outcomes of interest included speed, accuracy, and an overall speed accuracy tradeoff skill score. Normalized change in skill was assessed between baseline and retention on the trained (left) side. Speed was treated as total movement time (seconds). Error magnitude (cm) was measured as the summed length of the trace line outside the image boundary as calculated by a single researcher in ImageJ (Version 1.54c, Rasband, W.S., ImageJ, U. S. National Institutes of Health, Bethesda, MD, USA). To quantify how variations in precision and timing of motor performance jointly related to change in overall motor skill, a single skill value was calculated for each attempt using a speed–accuracy tradeoff function. Determination of the speed–accuracy tradeoff approximation function was based on a ten-subject, fixed-duration validation group as previously described by Johnson et al. (2022) [14]. Attention was given to the tradeoff model criteria defined by Reis et al. (2009) [24]. Using non-linear least squares analysis, the following monotonic function (Equation (1)) was selected, where *r* is error magnitude, *t* is moving time, and *a* and *b* are dimensionless-free parameters defined as “fixed” and “skill” values, respectively, based on mean percent change (i.e., 1.2% versus 143.8%) in pre- and post-test performance:

(1) $$r=\frac{a}{e^{bt}}$$

Equation (1) was solved for “skill” parameter (Equation (2)):

(2) $$b=\frac{\ln\left(a\right)-ln(r)}{t}$$

Healthy older adult motor outcomes were also compared, by practice group, to those of healthy younger adults on the same task using data reported previously by Johnson et al. (2022) [14]. Of note, Johnson et al. (2022) randomly assigned young adults to no-reactivation (control), one-trial-, three-trials-, or ten-trails-per-session groups after initial task learning [14]. In that study, young adults in the three-per-session (“brief reactivation”) group showed significantly greater transfer skill than those in the one-per-session and ten-per-session groups at two-week follow-up; no between-group differences were found between the one-per-session and ten-per-session groups in retention or transfer skill [14]. Accordingly, only two reactivation schedules (brief and long) were investigated here. However, all other aspects of study design including task instructions, session timing, and intertrial interval were held constant between studies [14].

To support meaningful age-related comparisons following the main study findings, ten older adults were randomly selected in a balanced manner from the brief and long practice groups to continue training until they achieved a within-session average skill score equal to or greater than the average retention score of healthy younger adults (M = 0.08). This continued training, up to seven additional sessions, took place after participants’ completion of an initial retention and transfer assessment at two-week follow-up. All additional sessions followed original training methods. Skill retention and transfer were then re-assessed in these ten subjects according to original testing methods.

RStudio (v2022.02.1+461 “Prairie Trillium”, RStudio Team 2020, PBC, Boston, MA, USA) was used to perform all statistical analyses. Levene’s tests were used to assess equality of variance and Shapiro–Wilk tests were used to assess the normality of the data. Skill scores were approximately normally distributed at all time points throughout the main study and continued practice. Movement time and error magnitude were not normally distributed in the main study data at several time points. Therefore, parametric statistical analysis was used for skill score comparison and non-parametric analyses were used for movement time and error magnitude comparison. Non-parametric analysis is also reported for the continued-practice group given the small size of the subset sample. All analyses were performed using a significance level of α= 0.05. To control for family-wise error rate, Bonferroni adjustments were made for planned multiple post hoc comparisons following a significant omnibus result.

## 3. Results

### 3.1. Healthy Older Adults

There were no significant between-group (i.e., brief versus long reactivation) differences in speed (Mann–Whitney U test, U = 217, *p* = 0.65; time (s) [M ± SD]: 3/session = 52.28 ± 29.04; 10/session = 53.85 ± 25.62), accuracy (Mann–Whitney U test, U = 189, *p* = 0.78; error (in) [M ± SD]: 3/session = 16.40 ± 29.04; 10/session = 11.90 ± 8.10), or skill (T-test, t = −0.09, *p* = 0.93; skill score [M ± SD]: 3/session = 0.01 ± 0.02; 10/session = 0.01 ± 0.02) among the older adult groups at baseline. A two-way analysis of variance (ANOVA) was used to assess skill by group and visit factors. Friedman’s tests were used to assess speed and accuracy by visit factor for each group. Pre-planned, post hoc, between-group comparisons were then made at Session 1 and Session 7 timepoints. The main findings of these comparisons were that older adults displayed similar acquisition, retention, and transfer skills regardless of the length of practice-induced reactivation. That is, the reactivation schedule did not appear to influence older adults’ motor learning outcomes in this task.

**Figure 2:**
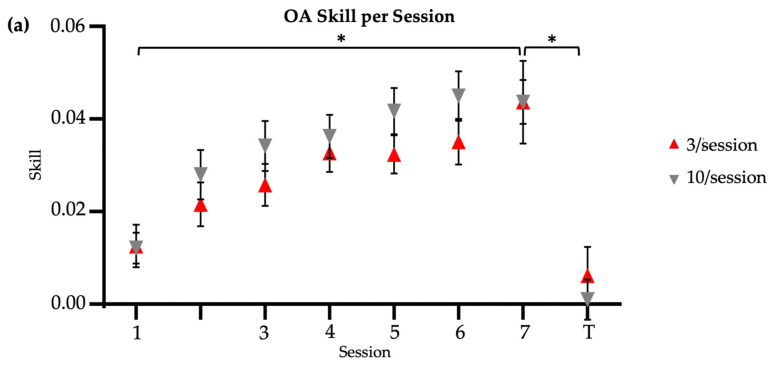

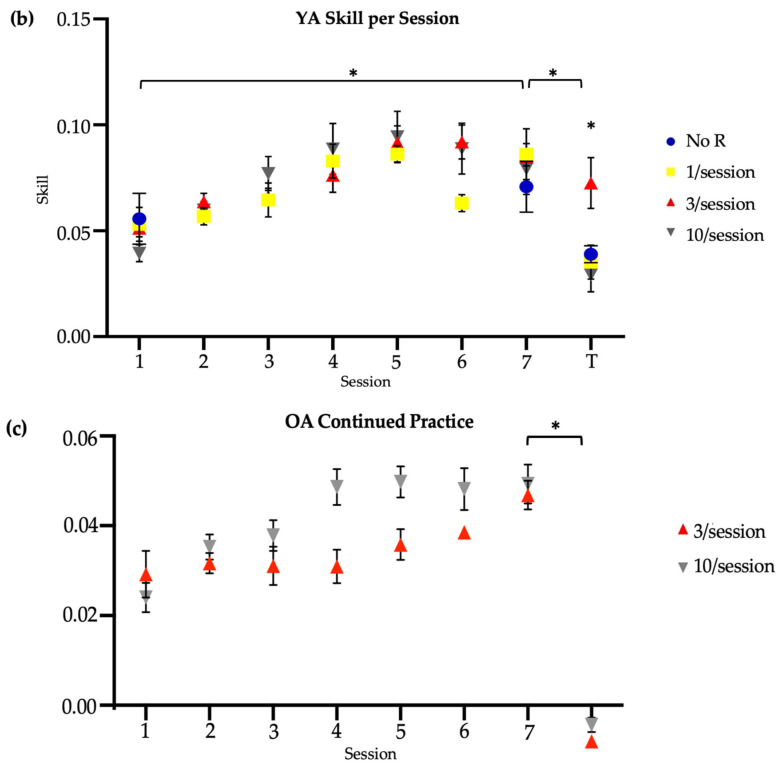
Skill score by session. * indicates significance at α= 0.05. Session 7 included retention (7) and transfer (T) tests. No R = no reactivation. (**a**) Older adults, main study. (**b**) Younger adults, reproduced from Johnson et al. (2022) [14]. (**c**) Older adults, continued practice.

Both brief and long reactivation older adult groups significantly improved their skill score from Session 1 to Session 7, with no significant between-group differences (two-way ANOVA [Session X Group]: main effect of session, F = 14.39, *p* < 0.01; main effect of group, F = 2.47, *p* = 0.12; session–group interaction, F = 0.55, *p* = 0.80) (Figure 2a). Time significantly decreased from Session 1 to Session 7 retention in both the 3/session (Friedman’s test, X^2^ = 46.52, *p* < 0.01) and 10/session (Friedman’s test, X^2^ = 30.17, *p* < 0.01) groups, with no significant between-group differences in retention speed at two-week follow-up (Mann–Whitney U test, U = 256, *p* = 0.13; time (s) [M ± SD]: 3/session = 31.56 ± 12.7; 10/session = 39.71 ± 17.04). Error magnitude significantly decreased from Session 1 to Session 7 retention in both the 3/session (Friedman’s test, X^2^ = 52.24, *p* < 0.01) and 10/session (Friedman’s test, X^2^ = 37.03, *p* < 0.01) groups, with no significant between-group differences in retention accuracy at two-week follow-up (Mann–Whitney U test, U = 194, *p* = 0.88; error (in) [M ± SD]: 3/session = 5.75 ± 4.57; 10/session = 6.35 ± 6.61). Regardless of the group, older adults also showed similar motor retention skill (t = −0.01, *p* = 0.99; skill score [M ± SD]: 3/session = 0.04 ± 0.02; 10/session = 0.04 ± 0.04) at two-week follow-up. Older adults showed similar between-group transfer speed (U = 206, *p* = 0.88; time (s) [M ± SD]: 3/session = 86.83 ± 59.56; 10/session = 83.92 ± 50.98), transfer accuracy (U = 202, *p* = 0.97; error (in) [M ± SD]: 3/session = 34.91 ± 37.36; 10/session = 29.50 ± 22.34), and transfer skill (t = −0.68, *p* = 0.50, Cohen’s d = 0.22; skill score [M ± SD]: 3/session = 0.006 ± 0.03; 10/session = 0.001 ± 0.02) at two-week follow-up. In summary, across the reactivation groups, older adults showed similar speed, accuracy, and skill for both the trained upper extremity (retention) and the untrained upper extremity (transfer) at two-week follow-up.

**Figure 3:**
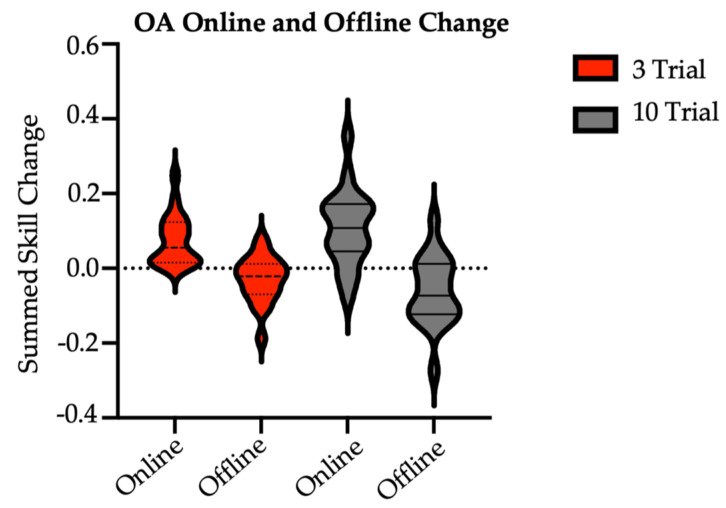
Sum of older adult online and offline skill changes with practice by group. OA = older adult (main study). There were no between-group differences at significance of α= 0.05. Online skill change was calculated as the difference in skill between the last trial and the first trial of a given session. Online values were summed over Session 1 through Session 7 retention. Offline skill change was calculated as the skill difference between the first and last trials of consecutive sessions. Offline values were summed over Sessions 1 through 7 retention.

Finally, there were no significant differences in the sum of online skill change (t = 1.26, *p* = 0.22; sum of skill [M ± SD]: 3/session = 0.07 ± 0.07; 10/session = 0.11 ± 0.10) or sum of offline skill change (t = −1.3, *p* = 0.20; sum of skill [M ± SD]: 3/session = −0.03 ± 0.06; 10/session = −0.06 ± 0.09) between the brief and long reactivation groups at two-week follow-up (Figure 3). Here, online skill change was calculated as the difference in skill between the last trial and the first trial of a given session. Offline skill change was calculated as the skill difference between the first trial of, e.g., Session 2 and the last trial of, e.g., Session 1. In short, across sessions, older adults in both groups made similar skill improvements during active task practice and showed similar stabilization (with a trend for slight performance declines) in post-practice periods.

### 3.2. Age-Related Differences

**Figure 4:**
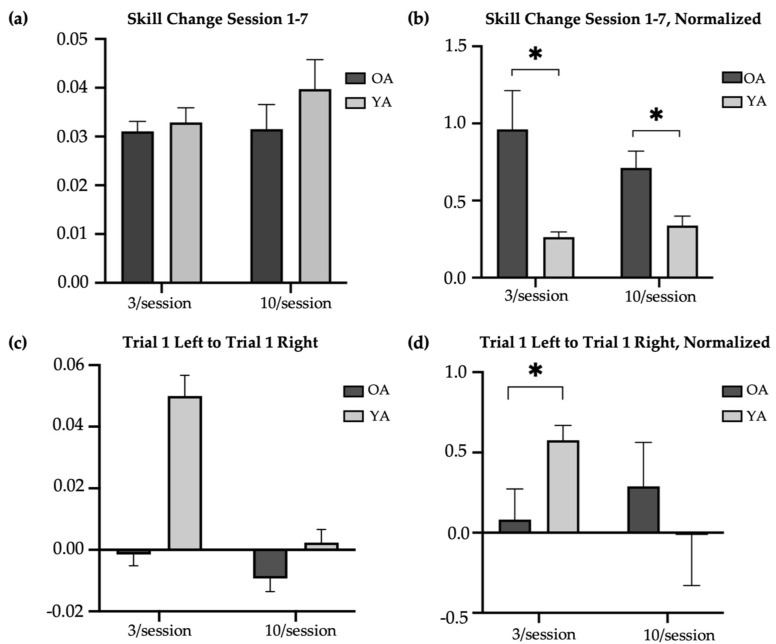
Age-related difference in change in skill with practice. * indicates significance at α= 0.05. OA = older adults (main study); YA = younger adults. (**a**) Difference in average raw skill in trained task from Session 1 (baseline) to Session 7 (retention) in older and younger adults. (**b**) Difference in average normalized skill in trained task from Session 1 (baseline) to Session 7 (retention) in older and younger adults. (**c**) Difference in first baseline trial (left) and first transfer trial (right) in older and younger adults. (**d**) Normalized difference in first baseline trial (left) and first transfer trial (right) in older and younger adults.

The older-adult skill scores were compared to the skill scores of younger adults reported by Johnson et al. (2022) (Figure 2b) [14]. Compared to older adults, younger adults had significantly higher raw skill scores at baseline in the 3/session (T-test, t = 5.14, *p* < 0.01; skill score [M ± SD]: younger = 0.05 ± 0.02; older = 0.01 ± 0.02) and 10/session (T-test, t = 4.07, *p* < 0.01; skill score [M ± SD]: younger = 0.04 ± 0.03; older = 0.01 ± 0.02) practice groups, at retention in the 3/session (T-test, t = −5.05, *p* < 0.01; skill score [M ± SD]: younger = 0.08 ± 0.03; older = 0.04 ± 0.02) and 10/session (T-test, t = −2.65, *p* = 0.01; skill score [M ± SD]: younger = 0.08 ± 0.04; older = 0.04 ± 0.02) practice groups, and at transfer in the 3/session (T-test, t = −4.43, *p* < 0.01; skill score [M ± SD]: younger = 0.07 ± 0.06; older = 0.006 ± 0.03) and 10/session (T-test, t = −3.29, *p* < 0.01; skill score [M ± SD]: younger = 0.03 ± 0.03; older = 0.001 ± 0.02) practice groups. However, there were no significant age-related differences in the change in trained (left) raw skill from Session 1 to Session 7 in the 3/session (T-test, t = 0.28, *p* = 0.78; change in raw skill score [M ± SD]: younger = 0.03 ± 0.02; older = 0.03 ± 0.01) or 10/session (T-test, t = 0.60, *p* = 0.55; change in raw skill score [M ± SD]: younger = 0.03 ± 0.02; older = 0.03 ± 0.03) practice groups (Figure 4a). Nevertheless, older adults in both the 3/session (Mann–Whitney U test, U = 94, *p* = 0.01; change in normalized skill score [M ± SD]: younger = 0.26 ± 0.19; older = 0.96 ± 1.59) and 10/session (Mann–Whitney U test, U = 93, *p* = 0.03; change in normalized skill score [M ± SD]: younger = 0.34 ± 0.35; older = 0.71 ± 0.68) practice groups showed significantly greater improvement in the trained skill over the two-week period when the change scores were normalized to baseline (the ratio of Session 1 to Session 7 skill difference and Session 1 to Session 7 skill product) (Figure 4b). This result likely reflects older adults having more opportunity to make performance gains, compared to younger adults, given their markedly poorer performance at baseline. On average, younger adults demonstrated their best performance on the trained (left) task during Session 5 in both the brief and long reactivation groups, whereas older adults demonstrated their best performance during Session 6 (10/session) and Session 7 (3/session). Younger adults showed a significantly larger difference in skill between their first baseline (left) trial and first transfer (right) trial than the older adults in the 3/session (T-test, t = 3.67, *p* < 0.05, Cohen’s d = 1.33; difference in skill score [M ± SD]: younger = 0.05 ± 0.05; older = −0.002 ± 0.03) but not 10/session (T-test, t = 1.14, *p* = 0.26; difference in skill score [M ± SD]: younger = 0.002 ± 0.03; older = −0.009 ± 0.03) practice groups (Figure 4c). The significance of this result did not change with normalization to baseline (Figure 4d).

### 3.3. Older Adults’ Continued Practice

With continued practice, only one older adult achieved an average within-session skill score equal to or greater than the average retention score of healthy younger adults (M = 0.08), and this was achieved in the sixth session. Therefore, all participants who continued practice attended seven additional sessions, including six sessions to train the task and one session to assess retention and transfer. Paired-sample Wilcoxon tests were used to identify whether continued practice resulted in a within-group improvement in skill for the trained task in the brief and long practice groups. Neither the brief (Wilcoxon test, V = 1, *p* = 0.125) nor long (Wilcoxon test, V = 0, *p* = 0.06) reactivation group showed significantly different left-handed skill between the new baseline and new retention time points (Figure 2c). Note, the new baseline was treated as the performance in the initial session of continued practice. Similarly, neither the brief (V = 12, *p* = 0.31) nor long (Wilcoxon test, V = 7, *p* = 0.99) practice group showed significantly different right-handed skill between the main study transfer and new transfer time points (Figure 2c).

Between the brief and long practice groups, there were no significant differences in new baseline speed (Mann–Whitney U test, U = 15, *p* = 0.69; time (s) [M ± SD]: 3/session = 41.10 ± 3.99; 10/session = 41.38 ± 13.40), new baseline accuracy (Mann–Whitney U test, U = 11, *p* = 0.84; error (in) [M ± SD]: 3/session = 8.17 ± 7.14; 10/session = 8.68 ± 5.57), or new baseline skill (Mann–Whitney U test, U = 14, *p* = 0.84; skill score [M ± SD]: 3/session = 0.03 ± 0.02; 10/session = 0.02 ± 0.01) in the older adults who continued practice. There were also no between-group differences in retention skill (Mann–Whitney U test, U = 13, *p* = 0.98; skill score [M ± SD]: 3/session = −0.01 ± 0.008; 10/session = −0.01 ± 0.004) measured prior to continued practice. Regardless of group, older adults showed similar retention speed (Mann–Whitney U test, U = 9, *p* = 0.55; time (s) [M ± SD]: 3/session = 35.02 ± 7.16; 10/session = 42.28 ± 16.15), retention accuracy (Mann–Whitney U test, U = 17, *p* = 0.42; error (in) [M ± SD]: 3/session = 4.05 ± 2.00; 10/session = 3.38 ± 1.96), and retention skill (Mann–Whitney U test, U = 12, *p* = 0.98; skill score [M ± SD]: 3/session = 0.05 ± 0.01; 10/session = 0.05 ± 0.02) in the seventh additional session. Similarly, there were no between-group differences in transfer speed (Mann–Whitney U test, U = 12, *p* = 0.99; time (s) [M ± SD]: 3/session = 96.34 ± 35.89; 10/session = 99.71 ± 37.31), transfer accuracy (Mann–Whitney U test, U = 16, *p* = 0.55; error (in) [M ± SD]: 3/session = 37.64 ± 11.08; 10/session = 35.56 ± 22.27), or transfer skill (Mann–Whitney U test, U = 9, *p* = 0.55; skill score [M ± SD]: 3/session = −0.008 ± 0.004; 10/session = −0.004 ± 0.007) with continued practice.

## 4. Discussion

The present study investigated the effect of brief versus long practice-induced reactivation schedules on the retention and intermanual transfer of a complex upper extremity motor task in healthy older adults. Our older-adult findings were compared to previously reported younger-adult data that were collected using a similar experimental design [14]. As hypothesized, three practice trials per session of a previously learned motor skill were sufficient to match the retention outcomes of a ten-trials-per-session condition at two-week follow-up. It appears that in older adults, like in younger adults, brief task reactivations are sufficient to strengthen and retain a motor skill. Against our hypothesis, intermanual transfer did not significantly benefit from brief compared to long practice in older adults; in this way, healthy older adult transfer performance notably differs from that of healthy younger adults for the same task [14]. These findings may suggest some efficacy in reducing the number of motor practice trials after initial skill learning, without compromising motor performance, in older adults; however, further evidence is needed to support this claim. If reproducible, this result would have clear implications for practice scheduling in physical rehabilitation, with the potential to limit practice-related fatigue and free up treatment time for additional interventions.

On the motor task used for this study, older adults were unable to achieve the level of proficiency demonstrated by younger adults, even when provided twice the amount of practice. However, skill improvement in the trained task over the two-week period did not significantly differ between age groups. These findings, that younger adults demonstrate overall higher skill at baseline and post-practice retention but a similar amount of skill change with practice, are consistent with previous studies. In an in-person version of the star-tracing task, Bootsma et al. (2021) reported that while older adults moved 73% slower than younger adults during the baseline phase, movement time improved similarly across both age groups from pre-test to post-test assessment [19]. In terms of motor accuracy, early evidence provided by Anshel (1978) demonstrated that although younger adults displayed superior performance at baseline, older adults had similar performance improvements with training [25]. Our results reinforce that, despite differences in raw scores, older and younger adults can show similar skill learning in a motor task over time.

Here, we expand on prior work by identifying age-related differences in the intermanual transfer of a complex motor skill following brief task reactivations. Age-related differences in task transfer following brief bouts of practice may be related to the discrepancies in the level of skill acquired for the trained task. It is worth noting that the average younger-adult skill score at baseline (M = 0.05) was higher than the average older-adult skill score at retention (M = 0.04). This difference, however, does not appear to be explained by a necessity for an extended practice period in older adults. After seven additional sessions, there remained no significant difference in intermanual transfer between brief and long reactivation conditions in a subset of the older adult sample. In fact, regardless of group, older adults who received additional practice did not significantly improve their performance in either the trained motor task or the transfer task. It could be that, given the complex task demands, older adults were unable to achieve a level of motor proficiency that supported transfer.

Task demand may have mediated skill discrepancies between older and younger samples through several mechanisms. The compensation-related utilization of neural circuits hypothesis (CRUNCH) suggests that as task demand increases, neural resources are recruited more rapidly in older than in younger adults to account for age-related neural inefficiencies (i.e., overactivation) [26]. It follows that, at a high level of task complexity, older adults reach a neural resource ceiling prior to younger adults, resulting in worse relative motor performance. Here, ceiling effects of compensatory neural recruitment in older, but not younger, adults may have resulted incomplete task acquisition (evidenced by markedly reduced raw motor performance) in the older sample, perhaps contributing to later impairments in skill transfer. Additionally, age-related impairments in specific sensorimotor processes including force modulation, upper extremity tool (e.g., computer mouse) use, and visuomotor rotation could have contributed to older adults’ diminished skill at all time points [27,28,29]. For example, speculatively, the older adult sample might have experienced greater difficulty making fine motor adjustments of the computer mouse than their younger counterparts on this motor task, resulting in both larger magnitudes of error and greater correction durations. Another possible explanation for our findings could be that older and younger adults differ in either ability or practice context required to reactivate a motor memory. Other considerations include age-related differences in the influence of conditions of practice on skill transfer. Constant as compared to variable practice schedules may interfere with skill transfer, and this effect may be exacerbated in healthy older adults. Note, our use of constant practice, despite evidence supporting the use of variable practice schedules in healthy adult populations, could be considered a limitation in our research design.

We acknowledge that limitations in our research design may have influenced the outcomes. The virtual nature of the task limited our control of the task environment including the surface height and handheld mouse properties. These variables were similarly uncontrolled for in the younger adult sample [14]. We were, however, able to enforce within-subject consistency in the task set-up, requiring that all sessions were completed using the same device. An additional limitation was the potential lack of precision in recording movement time. Movement time was measured manually by a single researcher and could be subject to variability in recorders’ reaction time. Finally, to match the younger-adult experimental design, we did not fix the intersession interval to a specific time; rather, we allowed for an intersession range of 24–72 h. Discrepancies in the intersession interval may influence the consolidation and offline learning of motor tasks.

Future studies should investigate underlying processes associated with age-related differences in motor skill transfer, including the use of neural monitoring methods, to identify targets for rehabilitation. Additionally, subsequent studies might examine age-related differences in reactivation-based motor learning using varied motor tasks and memory reactivation approaches. Future research should address the limitations of the present design by standardizing the motor-training environment and using more exact measures of movement timing and accuracy, perhaps through implementing an in-person design with kinematic tracking.

## 5. Conclusions

We found that in both younger and older adults, brief task-induced reactivation and subsequent reconsolidation of a motor trace are sufficient to retain motor performance in a complex upper extremity skill over a two-week period. However, brief practice benefits the intermanual transfer of a learned motor skill only in younger, not older, adults. Extending the overall practice period was not sufficient to address transfer impairments in a subset of our older adult sample. Age-related differences in the effect of brief practice on motor skill transfer likely relate to task demand. Age-related differences in ability or context required to reactivate a motor memory may also have contributed to findings. Reported skill discrepancies in older and younger adults may be attributed to motoric declines and neural inefficiencies with age. The findings have potential implications for motor training in sport, music, and rehabilitation including for physical and occupational therapies.

## Figures and Tables

**Table 1:** Older adult demographic data.

	3 Trials/Session	10 Trials/Session
n	20	20
Age (M yrs.)	71.7	72.1
Sex	17f/3m	12f/8m
SLUMS ^1^	27.9	28.1
MMSE ^2^	29.2	29.1

^1^ SLUMS: Saint Louis University Mental Status Exam. ^2^ MMSE: Mini-Mental State Examination.

**Table 2:** Session schedule.

Session	1	2	3	4	5	6	7	
							Retention	Transfer
	Trials	Trials	Trials	Trials	Trials	Trials	Trials	Trials
3/session	9	3	3	3	3	3	3	3
10/session	9	10	10	10	10	10	3	3

## Data Availability

The data that support the findings of this study are available from the corresponding author, K.P.W., upon reasonable request. The data are not publicly available due to participant consent.
